# Incidence and risk factors of adverse events after distal radius fracture fixation with volar locking plates: retrospective analysis of 2,790 cases

**DOI:** 10.2340/17453674.2024.42302

**Published:** 2024-11-18

**Authors:** Henri VASARA, Antti STENROOS, Petra TARKIAINEN, Anni AAVIKKO, Panu H NORDBACK, Turkka ANTTILA, Jussi KOSOLA, Samuli ASPINEN

**Affiliations:** 1 Department of Hand Surgery, Helsinki University Hospital and University of Helsinki, Helsinki; 2 Department of Orthopedics and Traumatology, Helsinki University Hospital, Helsinki; 3 Department of Surgery, Päijät-Häme Central Hospital, Lahti; 4 Department of Orthopedics and Traumatology, Kanta-Häme Central Hospital, Hämeenlinna, Finland

## Abstract

**Background and purpose:**

12–18% of patients encounter adverse events after distal radius fracture (DRF) surgery with volar locking plates (VLPs). Risk factors for which preventive measures could be administered are currently scarce. We aimed to examine the incidence of postoperative adverse events and assess the causes and risk factors for the adverse events after VLP fixation of DRFs.

**Methods:**

We performed a single-center retrospective cohort study evaluating all adult DRF patients treated with VLP fixation between 2009 and 2019 at Helsinki University Hospital. Patients with previous disabilities or ulnar fractures, other than styloid process fractures, in the affected extremity were excluded. We examined each patient’s treatment using the electronic medical records system and identified postoperative adverse events defined as any deviation from the ordinary postoperative course, showcasing clinical symptoms. We used multivariable binary logistic regression to assess the risk for adverse events.

**Results:**

2,790 cases of DRF were included. The incidence of adverse events was 16%. Hardware complications (8.3%), predominantly intra-articular screws (4.9%), were the most commonly encountered adverse events. Other frequent adverse events included carpal tunnel syndrome (2.8%), tendon complications (2.8%), and surgical site infections (1.5%). In the multivariable analysis, smoking, higher body mass index (BMI), alcohol abuse, C-type fractures, residual intra-articular displacement, and dorsal tilt were found as risk factors for adverse events.

**Conclusion:**

The incidence of adverse events was 16% after VLP fixation of DRFs. We identified several new risk factors for adverse events, which included residual dorsal tilt, intra-articular dislocation, insufficiently corrected inclination, smoking, alcohol abuse, and higher BMI.

Surgical treatment is carried out for 16–27% of all adult patients with distal radius fractures (DRFs), and the trend is increasing [1-3]. In recent years, fixation with volar locking plates (VLPs) has become the prevalent method of operative treatment for DRFs [2]. Although surgical treatment enables precise reduction and faster mobilization, and the absolute proportion of adverse events is less compared with conservative treatment, the risk of more severe adverse events is higher in operative treatment [4].

Although VLP fixation can be considered a common high-volume procedure, the current literature on risk factors consists of only a few studies. Previously published literature has identified C-type fractures, residual step-off, body mass index (BMI) ≥ 35, and patient age < 40 years (compared with > 40 years) to be risk factors for adverse events after VLP treatment of DRFs [5-8]. The impact of surgeon experience has had contradictory findings [6,9]. However, other independent studies have not confirmed these findings, and risk factors for which preventive measures could be administered are scarce.

We aimed to examine the incidence of postoperative adverse events after VLP fixation of DRFs. Furthermore, we assessed the causes and risk factors for the adverse events.

## Methods

### Study design and inclusion criteria

We conducted a retrospective single-center analysis of all surgically treated adult DRFs in Helsinki University Hospital between January 1, 2009 and December 31, 2019. The initial patient inquiry was done using NOMESCO procedure codes and the International Classification of Diseases-10 code S52.5. We included all DRFs with no previous deformity in the forearm. DRFs with ulnar styloid process (PSU) fractures were included, but all other ulnar fractures were excluded. Bilateral fractures were analyzed as separate cases. The study was reported according to STROBE guidelines.

### Data collection

The authors manually examined each patient’s data from the electronic medical records system. We acquired information on patient demographics, comorbidities, and associated injuries and examined all available fracture-related hospital visits. The patient records were available for a minimum period of 1 year postoperatively (median 5.9 years, IQR 3.2–8.6 years, range 1–11.4 years).

### Radiographic assessment

We assessed the standard pre- and postoperative posteroanterior and lateral wrist radiographs and intraoperative fluoroscopy images saved in the hospital’s Picture Archiving and Communications System for all DRF patients. Computed tomography images were assessed when available. All fractures were classified according to the AO/OTA classification into A, B, and C-type fractures, respectively [10]. In this study, we defined satisfactory postoperative fracture reduction as follows: dorsal tilt ≤ 5° [11], volar tilt ≤ 15°, inclination 10–30°, intra-articular diastasis or step-off ≤ 1 mm, and ulnar variance ≤ –2 mm [12].

### Fracture treatment

The surgeons were specialists or residents with a minimum of 3 years of surgical experience. In our institution, by protocol, hand surgeon specialists or residents operated on intra-articular (B and C type) fractures, and A-type fractures were operated on by orthopedic surgeon specialists or residents, respectively. The characteristics of the surgeons who performed the operations are presented in Table 1 (see Appendix).

**Table 1:** Characteristics of the surgeons who performed the operations

Factor	Hand surgeon specialists	Hand surgeon residents	Orthopedic specialists	Orthopedic residents	Other surgical residents	Total
Number of operations	742	1,538	55	418	37	2,790
Specialist surgeon present at the operation, n (%)	742 (100)	308 (20)	55 (100)	151 (36)	26 (70)	485 (46)
Number of surgeons	30	30	25	88	13	156 **^a^**
Number of operations per surgeon, median (IQR)	61 (37–94)	38 (56–72)	3 (2–5)	3 (2–6)	2 (1–2)	5 (2–19)

**^a^** The row total does not add up, as a single surgeon might have operated as both resident and specialist.

The patients were operated on a median of 8 days (IQR 5–13, range 0–30) after the initial injury. The fractures were operated on under fluoroscopy, and the treatment followed the AO guidelines [13]. All patients were operated on with either brachial plexus block or general anesthesia, and a tourniquet was used routinely. Prophylactic antibiotics were given to all patients during the anesthesia induction. 58% (n = 1,606) of the patients were treated with Acu-Loc 2 (Acumed LLC, Hillsboro, OR, USA) plate, 14% (n = 391) with DVR plate (Zimmer Biomet, Warsaw, IN, USA), 7% (n = 197) with Variable Angle LCP Two-column VDR Plate 2.4 (DePuy Synthes, Oberdorf, Switzerland), 8% (n = 278) with LCP Volar Column Distal Radius Plate 2.4 (DePuy Synthes, Oberdorf, Switzerland), 7% (n = 233) with Acu-Loc 1 plate (Acumed LCC, Hillsboro, OR, USA), and 3% (n = 85) with various miscellaneous plates, respectively.

A cast or a supportive bandage was placed postoperatively according to the surgeon’s preference. Based on the surgeon’s preference, mobilization without weightbearing was permitted immediately or after cast removal, usually at 2 or 5 weeks. 2,191 (76%) had a closed cast placed after the operation for a median length of 14 days (range 0–63 days). In addition, 1,088 (38%) patients had a removable cast placed for pain management.

Postoperatively, following a predetermined protocol, the fracture underwent its initial review at 2 weeks, conducted by either a physician or a nurse, during which the sutures were removed. All patients underwent a routine follow-up visit, including radiographs at the 5-week mark. Additional follow-up visits were scheduled if warranted in terms of possible adverse events or if the mobilization was not progressing at a normal rate. At the conclusion of the last scheduled follow-up visit, the patients were encouraged to contact our clinic with any issues or questions regarding the rehabilitation. The median duration of the clinical follow-up spanned 43 days (IQR 37–103 days, range 21 days to 7 years).

### Postoperative adverse events

Postoperative adverse events were the primary outcome of interest, and they were recorded and reviewed thoroughly. We defined an adverse event as any deviation from the ordinary postoperative course, showcasing clinical symptoms [14]. Adverse events related to preoperatively diagnosed associated injuries were not accounted for.

Surgical site infections were divided into superficial and deep infections based on the Centers for Disease Control and Prevention (CDC) criteria [15]. Mechanical complications included early reoperation due to unsatisfactory fixation or reduction, fixation failure, screws penetrating the dorsal cortex, and intra-articular screws. Flexor tendon adverse events are presented in detail in another publication [16]. Nerve complications were recorded if the nerve status differed between the preoperative and postoperative states. Carpal tunnel syndrome (CTS) was divided based on whether the patient needed an operation or resolved with conservative treatment. In addition, we recorded all nerve adverse events based on anatomy, functional disability (sensory, motor, or combined), and persistency (transient or persistent). Nonunion was defined as a fracture that had not achieved radiological and clinical union 12 months after the operation or if a secondary operation to enhance union was conducted. The union was considered delayed if full load bearing was not allowed 3 months after the operation. Complex regional pain syndrome (CRPS) was diagnosed according to Budapest criteria. If a patient only partially fulfilled the Budapest criteria, we classified it as other pain sensitization. Symptomatic post-traumatic arthritis presenting within 2 years of the operation was considered as an adverse event. Post-traumatic arthritis was diagnosed based on clinical symptoms and radiographic findings, but radiographs were not routinely screened for post-traumatic arthritis. Preoperative associated injuries, even if left undiagnosed during the initial evaluation, were not accounted for as adverse events. In addition, wrist ganglion cysts, trigger fingers, or Dupuytren’s contractures were not considered adverse events.

### Secondary operations

As a secondary outcome, we recorded all secondary operations and identified the underlying causes. Furthermore, we differentiated all postoperative adverse event-related secondary operations, as some secondary operations were due to preoperative associated injuries. All secondary operations were performed in the operation theater.

### Statistics

We obtained patient demographics and the number of adverse events using crosstabulations. We presented nominal values as counts and percentages. Continuous variables were presented as medians and interquartile range (IQR) or means and standard deviations (SD), depending on whether they followed the normal distribution. The normality of continuous variables was assessed visually by histograms and Q–Q plots and assessing the skewness value.

**Figure 1:**
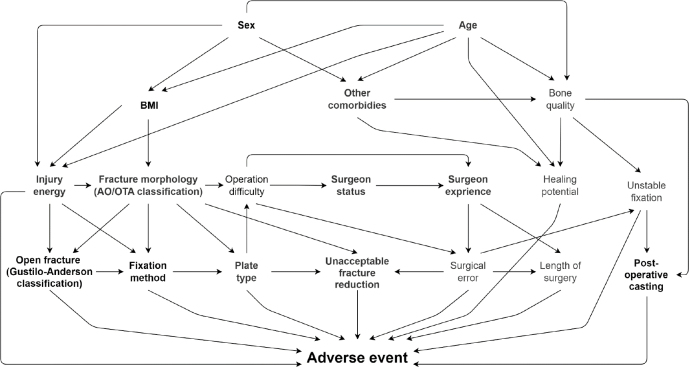
Directed acyclic graph used for determining confounding variables for covariate selection.

We used binary logistic regression to assess the risk factors for postoperative adverse events. We calculated odds ratios (OR) with 95% confidence intervals (CI) for each possible risk factor. In the case of missing variables, pairwise deletion was used. First, we performed a univariable analysis and selected potential risk factors for multivariable analysis. The covariables selected for adjustment were assessed with the help of a directed acyclic graph (DAG) we constructed (Figure 1, see Appendix), and DAGitty 3.0 software (available at https://dagitty.net released January 9, 2019) [17]. To examine whether we could include patients with simultaneous fractures in both forearms in our analysis, we compared their adverse event incidence with those who had unilateral fractures. Accordingly, to fulfill the basic assumptions of independent outcomes on logistic regression, we concluded the added adverse event risk as insignificant and assumed that the adverse event occurrence would be independent on each hand. The level of statistical significance was set as 5%. We used SPSS 29.0.0 (IBM Corp, Armonk, NY, USA, released November 17, 2022) for the statistical analysis.

### Ethics, data sharing plan, and conflicts of interest

We received the approval of the Helsinki University Hospital research committee to perform the study (HUS/234/2020). The research committee waived the requirement to seek ethical approval and informed patient consent based on Finnish legislation as the study did not involve patient interaction, and the patient data was pseudonymized for the analysis. Anonymized data is available based on reasonable request from the corresponding author. The authors declare no conflicts of interest. HV and SA utilized independent and institutional research funds. Complete disclosure of interest forms according to ICMJE are available on the article page, doi: 10.2340/17453674.2024.42302

## Results

**Figure 2:**
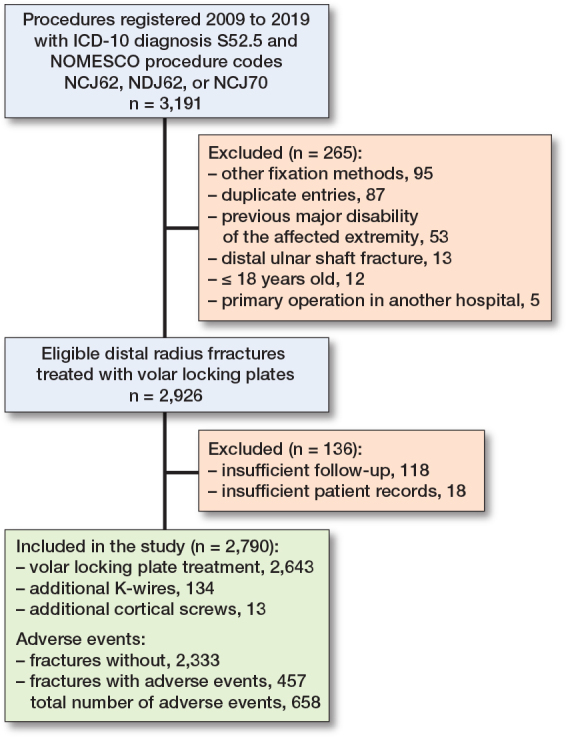
Patient inclusion flowchart.

We included 2,790 cases of distal radius fractures for the analysis. There were 100 patients with bilateral fractures, which were analyzed as separate cases (Figure 2).

The median age of the patients was 57 years (IQR 44–65), and 69% were female (Table 2). Overall, 134 patients had additional K-wires left in the surgery for fracture stabilization that were subsequently removed, and 13 had additional cortical screws. In 15% of the DRFs, the reduction of fractures in the postoperative radiographs was considered unsatisfactory.

**Table 2:** Patient characteristics. The categorical values are counts (%), if not otherwise specified

Patient demographics	
Age, median (IQR)	57 (44–65)
Female sex	1,930 (69)
Delay to surgery, days, median (IQR)	8 (5–13)
Associated injuries	
PSU fracture	1,738 (62)
SL-ligament injury	98 (3.4)
Acute CTS **^a^**	68 (2.4)
TFCC injury	63 (2.2)
Scaphoid fracture	31 (1.1)
Triquetrum fracture	16 (0.6)
Radiocarpal dislocation	4 (0.1)
Nerve injury **^b^**	9 (0.3)
LT-ligament injury	8 (0.3)
Other associated injuries **^c^**	11 (0.4)
Hand dominancy	
Dominant extremity	1,112 (40)
Non-dominant extremity	1,125 (40)
Bilateral injury	100 (4.3)
Not available	435 (16)
Injury energy **^d^**	
Low	2,030 (73)
Moderate	584 (21)
High	176 (6)
Comorbidities	
CCI, median (IQR)	1 (0–2)
Smoking **^e^**	366 (13)
Alcohol abuse **^f^**	140 (5)
Intravenous drug abuse	36 (1.3)
Diabetes	132 (4.7)
Type I	21 (0.8)
Type II, no insulin treatment	94 (3.4)
Type II, insulin treatment	17 (0.6)
Other considerable injuries **^g^**	276 (10)
BMI, mean (SD) **^h^**	26 (6)
Gustilo–Anderson classification	
Closed fracture	2,712 (97)
Open fracture	78 (2.8)
Type I	53 (1.9)
Type II	20 (0.7)
Type III	5 (0.2)
AO/OTA classification	
Type A	467 (17)
Type B	244 (8.7)
Type C	2,079 (75)

a Requiring a carpal tunnel release during the primary operation.

b Preoperative nerve injury requiring follow-up or interventions

c Other carpal bone fractures (n = 5) , extensor pollicis longus rupture (n = 4), perilunate injury (n = 2).

d Low-energy injury: equivalent to fall from standing height. Moderate energy injury: fall from 1–3 m, road traffic accident < 30 km/h, or equivalent. High energy injury: fall from > 3 m, road traffic accident > 30 km/h, or equivalent.

e Only current smoking included.

f Patients having multiple (≥ 2) alcohol-related emergency department visits, previous ICD-10 diagnoses referring to alcohol abuse, or alcohol-related end-organ diseases, such as liver cirrhosis or chronic pancreatitis.

g Including fractures and ligament injuries not included in the associated injuries, and traumatic brain and internal organ injuries.

h There were 849 missing values.

CCI = Charlson Comorbidity Index, CTS = carpal tunnel syndrome, LT = lunotriquetral, PSU = ulnar styloid process, SL = scapholunate, TFCC = triangular fibrocartilage complex.

### Adverse events

**Figure 3:**
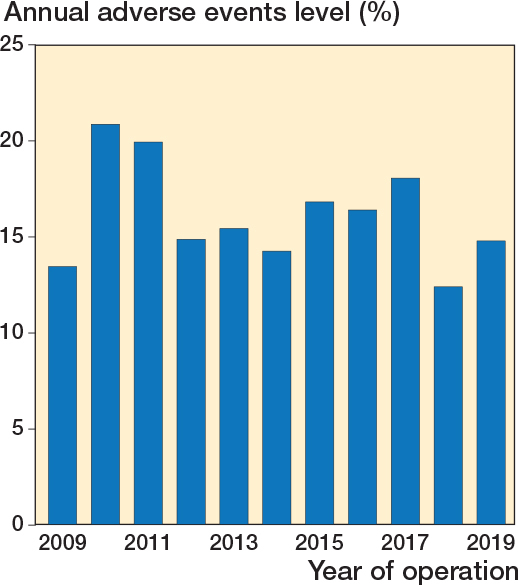
Proportional incidence of adverse events during the study period.

16% (n = 457) of DRFs sustained a total of 658 adverse events (Table 3). The yearly incidence varied between 12% and 21%, but there was no distinctive trend between the years (Figure 3). Adverse events categorized by AO/OTA fracture types showed that the adverse event incidence was higher for type B (15%) and C (18%) fractures when compared with type A fractures (11%), especially regarding mechanical adverse events. (Table 4, see Appendix).

**Table 3:** Postoperative adverse events. The presented values are counts (%). A single patient might present multiple adverse events

Patients with postoperative adverse events	457 (16)
Postoperative adverse events, total	658
Mechanical adverse events	232 (8.3)
Early reoperation due to malreduction	29 (1.0)
Intra-articular screws	138 (4.9)
Fixation failure	74 (2.7)
Screws penetrating the outer cortex	34 (1.2)
Nerve adverse events	89 (3.1)
CTS, transient	38 (1.4)
CTS, requiring operation	39 (1.4)
Median nerve injury, non-CTS	8 (0.3)
Ulnar nerve injury	3 (0.1)
Radial nerve injury	3 (0.1)
Tendon adverse events **^a^**	78 (2.8)
EPL rupture	17 (0.6)
EPL tendinopathy	6 (0.2)
Other extensor tendinopathies	13 (0.5)
Flexor tendinopathy	33 (1.2)
Flexor rupture	4 (0.1)
De Quervain	7 (0.3)
APL-rupture	1
Delayed union and nonunion **^b^**	71 (2.5)
Delayed union	65 (2.3)
Nonunion	6 (0.2)
Surgical site infections	42 (1.5)
Superficial infection	29 (1.0)
Deep infection	13 (0.5)
Pain sensitization **^c^**	38 (1.4)
CRPS I	18 (0.6)
CRPS II	4 (0.1)
Other pain sensitization	16 (0.6)
Other adverse events	
Post-traumatic arthritis **^d^**	32 (1.1)
Unspecified pain or stiffness **^e^**	16 (0.6)
Arterial injury	7 (0.3)
Compartment syndrome	3 (0.1)

a Tendinopathy: Physician-confirmed tendinitis, swelling, or pain moving the tendon in question.

b Nonunion: No achieved radiographic and clinical union 12 months after the operation or if a secondary operation to enhance union was conducted. The union was considered delayed if full load-bearing was not allowed 3 months after the operation.

c Complex regional pain syndrome (CRPS) was diagnosed according to Budapest criteria. If a patient only partially fulfilled the Budapest criteria, we classified it as other pain sensitization.

d Early onset post-traumatic osteoarthritis diagnosed within 2 years from the operation.

e Treated with plate removal.

APL = abductor pollicis longus. CRPS = Complex regional pain syndrome, CTS = Carpal tunnel syndrome, EPL = Extensor pollicis longus.

**Table 4:** Postoperative adverse events categorized by AO/OTA fracture types. The presented values are counts (%). A single patient might present multiple adverse events

Factor	Type A fractures (n = 464)	Type B fractures (n = 242)	Type B fractures (n = 2,052)
Patients with postoperative adverse events	53 (11)	36 (15)	368 (18)
Postoperative adverse events, total	72	44	542
Mechanical adverse events	22 (4.7)	16 (6.6)	194 (9.5)
Early reoperation due to malreduction	2 (0.4)	2 (0.8)	25 (1.2)
Intra-articular screws	11 (2.4)	5 (2.1)	122 (5.9)
Fixation failure	4 (0.9)	9 (3.7)	61 (3.0)
Screws penetrating the outer cortex	7 (1.5)	2 (0.8)	25 (1.2)
Nerve adverse events	4 (0.9)	10 (4.1)	75 (3.7)
CTS, transient	1 (0.2)	7 (2.9)	30 (1.5)
CTS, requiring operation	2 (0.4)	3 (1.2)	34 (1.7)
Median nerve injury (no CTS)	1 (0.2)	0 (0.0)	7 (0.3)
Ulnar nerve injury	0 (0.0)	0 (0.0)	3 (0.1)
Radial nerve injury	0 (0.0)	0 (0.0)	3 (0.1)
Tendon adverse events **^a^**	12 (2.6)	6 (2.5)	60 (2.9)
EPL rupture	3 (0.6)	1 (0.4)	13 (0.6)
EPL tendinopathy	0 (0.0)	1 (0.4)	5 (0.2)
Other extensor tendinopathies	3 (0.6)	1 (0.4)	9 (0.4)
Flexor tendinopathy	3 (0.6)	3 (1.2)	27 (1.3)
Flexor rupture	0 (0.0)	0 (0.0)	4 (0.2)
De Quervain	3 (0.6)	1 (0.4)	3 (0.1)
APL rupture	0 (0.0)	0 (0.0)	1 (0.0)
Delayed union and nonunion **^b^**	13 (2.8)	2 (0.8)	56 (2.7)
Delayed union	13 (2.8)	2 (0.8)	50 (2.4)
Nonunion	0 (0.0)	0 (0.0)	6 (0.3)
Surgical site infections	5 (1.1)	3 (1.2)	34 (1.7)
Superficial infection	4 (0.9)	2 (0.8)	23 (1.1)
Deep infection	1 (0.2)	1 (0.4)	11 (0.5)
Pain sensitization **^c^**	7 (1.5)	1 (0.4)	30 (1.5)
CRPS I	2 (0.4)	1 (0.4)	15 (0.7)
CRPS II	2 (0.4)	0 (0.0)	2 (0.1)
Other pain sensitization	3 (0.6)	0 (0.0)	13 (0.6)
Other adverse events			
Post-traumatic arthritis **^d^**	0 (0.0)	3 (1.2)	31 (1.5)
Unspecified pain or stiffness **^e^**	3 (0.6)	0 (0.0)	13 (0.6)
Arterial injury	4 (0.9)	0 (0.0)	3 (0.1)
Compartment syndrome	0 (0.0)	0 (0.0)	3 (0.1)

a-e See Table 3.

**Figure 4:**
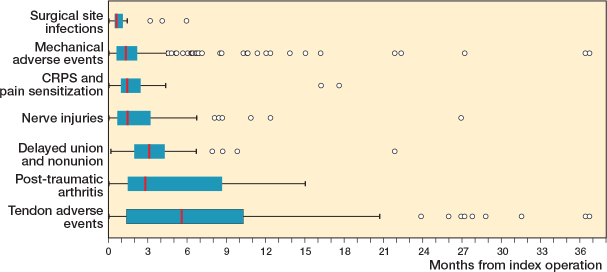
Box-plot figure representing the time from the VLP fixation of DRFs to the diagnosis of the adverse events. There was 1 outlier patient, a tendon adverse event at 45 months. The vertical line represents the median, the edges of the box represent lower and upper quartile values (IQR), the whiskers represent minimum and maximum data values (1.5 IQR values), and the circles represent outlier values.

67% of the adverse events were diagnosed during the first 3 months postoperatively. These included surgical site infections, most mechanical adverse events, pain sensitizations including CRPS, and nerve injuries. Subsequently, 24% of adverse events were diagnosed between 3 and 12 months postoperatively. Late-onset adverse events (9%), diagnosed after 1 year postoperatively, were typically tendon-related issues, and individual cases of mechanical adverse events, typically related to tendon irritation (Figure 4).

2.8% of DRFs developed carpal tunnel syndrome (CTS) postoperatively, of which half required a carpal tunnel release. In addition, 0.5% (n = 14) had other postoperative nerve lesions (8 affecting the median nerve, 3 radial nerve, and 3 ulnar nerve). 10 of these were completely sensory lesions, whereas 1 affected motor functions, and 3 were combined.

Overall, 2.8% (n = 78) of DRFs had tendon complications after the VLP fixation. The suspected cause was the VLP in 36 cases, screws penetrating the dorsal cortex in 21 cases (either extensor pollicis longus [EPL], other extensors, or de Quervain tendinopathies), fracture fragments in 2 cases, and scar formation in 2 cases. In 17 patients, the reason remained unknown. Individual screws were removed in 8 cases, and the whole VLP was removed in 52 cases. 17 cases had an EPL tendon rupture, 4 of which had screws penetrating the dorsal cortex.

Surgical site infection was diagnosed in 1.5% (n = 42) of the DRFs. 29 of these were superficial infections and healed with nonoperative measures (removing stitches, drainage, and oral antibiotics). However, 0.5% (n = 13) of the cases had a deep surgical site infection, of which 9 underwent revision surgery due to the infection. Only a single case with a deep infection had sustained a type I open fracture. All other cases of deep surgical site infections had closed fractures.

8.3% (n = 232) of the DRFs had mechanical complications. 1.0% (n = 29) of the cases required an early refixation median 5 (range 0–21) days after the primary operation due to unsatisfactory fracture reduction, early fixation failure, or screw-related problems. During the follow-up, 4.9% (n = 138) of the DRFs were diagnosed with intra-articular screws, 22 of which also sustained a fixation failure. In addition, there were 29 DRFs with screws penetrating the dorsal cortex, none of which had sustained fixation failure. Overall, fixation material loosening or fracture reduction failure was seen in 2.7% (n = 74) of cases during follow-up without significant new injuries. Type C fractures in particular (5.9%, n = 122/2,052) had intra-articular screws more frequently than A (2.4%, n = 11/464) or B (2.1%, n = 5/242) type fractures (Table 4, see Appendix).

10% (n = 292) of the DRFs required secondary operations after VLP fixation due to adverse events (Table 5). Including secondary operations due to associated injuries, overall 11% (n = 339) of the DRFs had at least 1 secondary operation (range 1–5). The most common secondary operation was plate removal, which was done for 5.6% (n = 155) of the DRFs due to an adverse event, and for 5.9% (n = 165) including also plate removals due to treatment of associated injuries.

**Table 5:** Secondary operations resulting from adverse events after VLP fixation of DRFs. A single patient might present multiple secondary operations in different categories

Patients with secondary operations	292 (10)
Secondary operations, total	374
Material removal	238 (8.5)
Plate removal	155 (5.6)
Screw removal	83 (3.0)
Early refixation	30 (1.1)
Carpal tunnel release	39 (1.4)
Arthroscopy	13 (0.5)
Tendon reconstruction	16 (0.6)
EIP pro EPL transposition	13 (0.5)
EIP pro EDC2 transposition	1
EPL reconstruction	1
FPL reconstruction	1
Arthrodesis	12 (0.4)
Radioscapholunate arthrodesis	8 (0.3)
Total wrist arthrodesis	4 (0.1)
Osteotomy	9 (0.5)
Darrach procedure	4 (0.1)
Ulnar shortening	3 (0.1)
Radius corrective osteotomy	2 (0.1)
Revision due to infection a	9 (0.3)
Fasciotomy	3 (0.1)
Nonunion surgery	1

a 2 patients had 3 operations, whereas the other 7 had 1.

EDC= extensor digitorum communis, EIP = extensor indicis propria, EPL = extensor pollicis longus, FPL = flexor pollicis longus.

### Risk factors for adverse events

Age, sex, diabetes, hand dominancy, presence of PSU fractures, and surgeon status or operation count did not have a statistically significant association with the incidence of adverse events, and therefore they were not accounted for in the multivariable models (Table 6). Prolonged closed casting time was strongly associated with more adverse events. However, we could not build a model considering the necessary confounding factors, such as fixation stability.

**Table 6:** Risk of postoperative adverse events and univariable binary logistic regression analysis for patients operated on with VLP. Variables are binominal, and the non-predisposed group is used as a reference if not otherwise specified

Factor	Risk of adverse events **^a^** (%)	Odds ratio (CI)
Age, years		
18–40	72/552 (13)	Reference
40–65	271/1,515 (18)	1.5 (1.1–1.9)
65–75	93/595 (16)	1.2 (0.9–1.7)
> 75	21/128 (16)	1.3 (0.8–2.2)
Additional fixation procedures		
Additional K-wires	49/134 (37)	3.2 (2.2–4.6)
Additional cortical screws	4/13 (31)	2.3 (0.7–7.4)
Patient demographics		
Male sex	156/860 (18)	1.2 (1.0–1.5)
CCI (+1) **^b^**		0.99 (0.92–1.06)
BMI (+ 1) **^b^**		1.03 (1.01–1.05)
Diabetes	24/132 (18)	1.1 (0.7-1.8)
Alcohol abuse	37/140 (26)	1.9 (1.3–2.8)
Active smoking	74/365 (20)	1.4 (1.0–1.8)
Drug abuse	12/36 (33)	2.6 (1.3–5.2)
Hand dominancy		
Dominant extremity	195/1,112 (18)	Reference
Non-dominant extremity	176/1,125 (16)	0.9 (0.7–1.1)
Both extremities	15/100 (15)	0.8 (0.5–1.5)
Injury energy		
Low	316/2,030 (16)	Reference
Moderate	103/584 (18)	1.2 (0.9–1.5)
High	38/176 (22)	1.5 (1.0–2.2)
AO/OTA classification		
Type A	53/467 (11)	Reference
Type B	36/244 (15)	1.4 (0.9–2.1)
Type C	368/2,079 (18)	1.7 (1.2–2.3)
Gustilo–Anderson classification		
Closed fracture	438/2,712 (16)	Reference
Type I	11/53 (21)	1.4 (0.7–2.7)
Type II	7/20 (35)	2.8 (1.1–7.0)
Type III	1/5 (20)	1.3 (0.1–12)
PSU fracture		
No PSU fracture	175/1050 (17)	Reference
PSU base fracture	101/550 (18)	1.1 (0.9–1.5)
PSU tip fracture	180/1,188 (15)	0.9 (0.7–1.1)
Insufficient operative fracture reduction **^c^**		
Insufficient reduction, any	138/412 (34)	3.2 (2.6–4.1)
Residual intra-articular dislocation (+1 mm) **^b^**	1.6 (1.4–1.8)	
Residual dorsal tilt (+1°) **^b^**		1.07 (1.03–1.12)
Residual radial shortening ≥ 2mm	17/74 (23)	1.5 (0.9–2.7)
Residual inclination ≥ 30°	7/13 (54)	6.0 (2.0–18)
Residual Inclination ≤ 10°	5/9 (56)	6.4 (1.7–24)
Postoperative casting		
Closed casting time (+1 week) **^b^**		1.3 (1.2–1.3)
Removable casting time (+1 week) **^b^**		1.09 (1.03–1.16)
Surgeon status **^d^**		
Hand surgery specialist	133/744 (18)	Reference
Hand surgery resident	253/1,536 (17)	0.9 (0.7–1.1)
Orthopedic specialist	10/55 (18)	1.0 (0.5–2.1)
Orthopedic resident	58/418 (14)	0.7 (0.5–1.0)
Other resident	3/37 (8)	0.4 (0.1–1.3)
Procedure count (+1 procedure) **^b^**		1.0 (1.0–1.0)

a Number of adverse events/predisposed.

b Analyzed as a continuous variable assuming linear risk increase.Each increase in the unit indicated in parentheses increases the risk by the factor of the OR.

c Determined from either intraoperative radiographs or postoperative radiographs taken directly after the operation.

By protocol, A-type fractures were operated on by orthopedic surgeons, whereas B- and C-type fractures were operated on by hand surgeons. For abbreviations, see Table 2.

BMI, alcohol and drug abuse, and smoking were significant risk factors for adverse events regarding patient demographics (Table 7). Patients with high-energy injuries and C-type fractures had a significantly greater risk of adverse events. In addition, residual dorsal tilt, intra-articular dislocation, and insufficiently corrected inclination were also significant risk factors for postoperative adverse events.

**Table 7:** Multivariable binary logistic regression for adjusted odds ratios (OR) for adverse events after VLP fixation

Factor	Adjusted OR (CI)
Patient demographics	
BMI (+ 1) **^a^**	1.02 (1.01–1.04)
Alcohol abuse	1.8 (1.2–2.7)
Active smoking	1.4 (1.0–1.8)
Drug abuse	2.7 (1.3–5.5)
Additional K-wires	2.9 (2.0–4.2)
Injury energy **^b^**	
Low	Reference
Moderate	1.3 (1.0–1.8)
High	2.0 (1.3–3.2)
AO/OTA classification	
Type A	Reference
Type B	1.3 (0.8–2.0)
Type C	1.6 (1.2–2.2)
Gustilo–Anderson classification	
Closed fracture	Reference
Type I	1.3 (0.6–2.5)
Type II	2.5 (1.0–6.3)
Type III	1.0 (0.1–9.1)
Insufficient operative fracture reduction **^c^**	
Insufficient reduction, any	3.0 (2.4–3.8)
Residual intra-articular dislocation (+1 mm) **^a^**	1.6 (1.4–1.7)
Residual dorsal tilt (+1°) **^a^**	1.07 (1.02–1.11)
Residual radial shortening ≥ 2 mm	1.4 (0.8–2.4)
Residual inclination ≥ 30°	5.6 (1.9–17)
Residual inclination ≤ 10°	5.7 (1.5–22)

a Analyzed as a continuous variable assuming linear risk increase. Each increase in the unit indicated in parentheses increases the risk by the factor of the OR.

b Low-energy injury: equivalent to fall from standing height. Moderate-energy injury: fall from 1–3 m, road traffic accident < 30 km/h, or equivalent. High-energy injury: fall from > 3 m, road traffic accident > 30 km/h, or equivalent.

c Determined from either intraoperative radiographs or postoperative radiographs taken directly after the operation.

Adjusted covariates: BMI, alcohol abuse, smoking, and diabetes: age and sex.

Injury energy: age, sex, and BMI.

AO/OTA classification: age, sex, injury energy.

Gustilo–Anderson classification and additional K-wires: age, sex, fracture energy, AO/OTA-classification.

Anatomical reduction: age, sex, AO/OTA-classification, plate type.

## Discussion

We aimed to examine the incidence of postoperative adverse events after VLP fixation of DRFs. Furthermore, we assessed the causes and risk factors for the adverse events.

We found an adverse event incidence of 16% after surgical treatment of DRF with VLP and a secondary operation incidence of 10%. Alcohol and drug abuse, residual dorsal tilt, and insufficiently corrected inclination were established as novel risk factors for adverse events. In addition, our findings confirm that type C-fractures, smoking, higher BMI, and intra-articular dislocation are risk factors for adverse events.

The present study is so far the most extensive single-center study in the literature, reporting numerous previously unreported risk factors for postoperative adverse events after VLP fixation for DRFs.

The incidence of adverse events in our study was consistent with the previous literature. Recent studies have reported adverse event incidences between 12 and 18% [5-7,18-20]. Although the definitions used for adverse events and complications vary, the most common adverse events seem well established. Overall, the most common adverse event appears to be CTS, followed by hardware-related problems. Union rates after operative treatment for DRFs are high, and delayed union or nonunion is rarely encountered. CRPS and nerve lesions remain infrequent but significant adverse events. Furthermore, 5–10% of patients undergo a secondary operation, the majority being plate removal [5-8,19,20].

The incidence of mechanical complications was considerably high in our cohort, accounting for a total of 8.3% of the patients. Intra-articular screws especially were a prominent adverse event with an incidence of 4.9%. In the previous literature, similarly high incidences of hardware-related adverse events have been reported only by DeGeorge et al. [5], whereas other comparable cohorts have reported an intra-articular screw penetration incidence of 1.0–1.5% [6,9,18,19]. We consider the difference to be partly explained due to differences in fracture morphology, as we had 75% AO type C fractures, whereas the cohort reported by DeGeorge et al. had 67% [5], compared with 49–64% in other publications [6,9,18,19]. However, most of these adverse events undeniably could have been avoided with more meticulous planning and execution in addition to fluoroscopic imaging.

Multi-fragmented intra-articular (AO C-type) fractures have previously been associated with a higher rate of adverse events, although the current evidence has not been unanimous [7-9,19]. Our analysis showcased an approximately 60% increase in risk for adverse events for type C fractures when compared with A-type fractures. Considering the overall evidence, type C fractures can be regarded as an established risk factor for adverse events, whereas A- and B-type fractures have a comparable incidence for adverse events.

Residual intra-articular dislocation, dorsal tilt, and abnormal inclination were found as independent predictors of adverse events. In addition, our results revealed that combining VLP fixation with K-wires was associated with a higher risk of adverse events. This emphasizes that aiming for anatomic restoration has value, but the routine use of additional K-wires should always be rationalized as an exception instead of an arbitrary addition.

### Limitations

The main limitations of our study are due to its retrospective nature. First, the clinical follow-up for the majority of the patients was brief. However, as we demonstrated, the vast majority of adverse events are diagnosed during the first year. We believe excluding the patients with short clinical follow-up would have caused selection bias, as most patients who recovered well had only one routine outpatient follow-up visit. In addition, the patients had a low threshold to seek re-evaluation due to symptoms, and as the only public hospital in the region focusing on forearm, wrist, and hand fracture surgery, we have an excellent re-referral rate if other physicians diagnosed adverse events. Second, patient-reported outcomes were not available. Therefore, the clinical impact of the adverse events is not entirely well established.

### Conclusions

The incidence of adverse events after DRF surgery with VLP fixation was 16%. AO type C-fractures, residual dorsal tilt, intra-articular dislocation, insufficiently corrected inclination, smoking, alcohol abuse, and higher BMI were shown as risk factors for adverse events. In perspective, our study provides extensive novel information regarding injuries associated with DRFs and postoperative adverse events. Attention should be given to plate and especially screw placement during operations as it is the most distinct way to reduce adverse events and secondary operations.

## Notes

SA, AS, JK, PN, TA, and HV planned the study. HV, AS, PT, AA, PN, TA, and SA collected the data. HV performed the statistical analyses and wrote the initial manuscript. All authors contributed to the interpretation of the results, participated in manuscript revisions, and accepted the final manuscript.

Acknowledgement – Open access was funded by Helsinki University Library.

Handling co-editor: Taco Gosens

*Acta* thanks anonymous reviewers for help with peer review of this manuscript.
